# Effect of Feed Additives as Alternatives to In-feed Antimicrobials on Production Performance and Intestinal *Clostridium perfringens* Counts in Broiler Chickens

**DOI:** 10.3390/ani10020240

**Published:** 2020-02-03

**Authors:** Silje Granstad, Anja B. Kristoffersen, Sylvie L. Benestad, Siri K. Sjurseth, Bruce David, Line Sørensen, Arnulf Fjermedal, Dag H. Edvardsen, Gorm Sanson, Atle Løvland, Magne Kaldhusdal

**Affiliations:** 1 Norwegian Veterinary Institute, P.O. Box 750 Sentrum, 0106 Oslo, Norway; anjab@ifi.uio.no (A.B.K.); sylvie.benestad@vetinst.no (S.L.B.); siri.sjurseth@vetinst.no (S.K.S.); magne.kaldhusdal@vetinst.no (M.K.); 2 Nortura SA, P.O. Box 360 Økern, 0513 Oslo, Norway; robert.bruce.david@nortura.no (B.D.); atle.lovland@nortura.no (A.L.); 3 Felleskjøpet Fôrutvikling AS, Nedre Ila 20, 7018 Trondheim, Norway; line.sorensen@fkf.no (L.S.); gorm.sanson@fkf.no (G.S.); 4 Fiskå Mølle AS, Fiskåvegen 1010, 4120 Tau, Norway; arnulf.fjermedal@fiska.no; 5 Norgesfôr AS, Torggata 10, 0181 Oslo, Norway; dag.henning.edvardsen@norgesfor.no

**Keywords:** broilers, feed additives, probiotics, prebiotics, phytogenics, organic acids, anticoccidials, necrotic enteritis, *Clostridium perfringens*, production performance

## Abstract

**Simple Summary:**

For many years, antibiotics were added to chicken feed to prevent disease and promote growth. This practice has been banned or voluntarily abolished in many countries. However, most countries still allow the use of in-feed ionophorous coccidiostats, which are drugs that possess both antiparasitic and antibacterial properties. Concerns related to antimicrobial resistance have led to increased focus on broiler chickens raised without the use of any antimicrobial agents, and the interest in non-antibiotic feed additives with beneficial effects on gastrointestinal health and productivity is growing. In this study, feed additives with active components belonging to the product classes probiotics, prebiotics, phytogenics and/or organic acids were assessed for their effect on intestinal health and production performance in broiler chickens. Collectively, the group of non-antibiotic feed additives improved gut health and performance, but not to the same extent as the ionophorous coccidiostat narasin. Probiotics and prebiotics had the overall best performances during coccidia challenge, phytogenics improved overall feed conversion and reduced counts of the intestinal bacterium *Clostridium perfringens*, and organic acids increased weight gain independent of age. This study provides comparable and unbiased results from testing of alternatives to antibiotics in a uniform experimental model highly relevant to commercial conditions.

**Abstract:**

Numerous non-antibiotic feed additives (alternatives to antibiotics, ATAs) have been marketed, but few have been evaluated under uniform testing conditions modelling commercial flocks. We compared 24 ATA treatments and the ionophorous coccidiostat narasin against a diet without any feed additives. Feed conversion ratio and body weight gain were registered from day 0 to 28 in Ross 308 chickens housed on litter floor. The chickens were challenged with *Eimeria* spp., and cecal *Clostridium perfringens* (CP) counts were investigated. Active components from all ATA classes had a positive impact on intestinal health or production performance. Whereas narasin had a strong CP-reducing effect in combination with performance-promoting impact, only two ATA treatments achieved significantly beneficial effects on CP counts as well as feed conversion during the time span following *Eimeria* challenge. Active components present in these two treatments include a *Bacillus subtilis* probiotic strain, short- and medium-chain fatty acids and *Saccharomyces cerevisiae* components. Different ATA classes had beneficial impact during distinct rearing phases and on specific performance targets, suggesting that optimizing combinations and use of active components can make ATAs even more useful tools in broiler rearing without the use of in-feed antimicrobials. Further studies of promising ATAs and ATA combinations are required.

## 1. Introduction

The use of antimicrobial growth promoters (AGPs) was abolished in Sweden, Norway and Denmark in 1986, 1995 and 1998–1999, respectively [1]. As a response to this development, the use of ionophorous coccidiostats (e.g., narasin) in broiler feeds increased and became more important than before [2]. In 2006, the European Union implemented a total ban of AGPs, meaning that antimicrobials other than coccidiostats and histomonostats were no longer allowed as feed additives in the poultry industry [3,4]. Coccidiostats like narasin and other ionophores are still approved in the European Union for control of coccidiosis caused by the parasitic protozoans *Eimeria* spp. in poultry.

Ionophores are primarily approved for control of coccidiosis but may also have antibacterial and antiviral properties [5]. Narasin has a well-known inhibitory effect on the potential pathogen *Clostridium perfringens* (CP), which is associated with the intestinal disease necrotic enteritis (NE) in broiler chickens [6,7]. Selected ionophores have been suggested as novel antimicrobial agents to control infectious diseases in animals as alternatives to antimicrobial classes used to treat human disease [8]. Furthermore, concerns have been raised regarding the possibility that the use of narasin and other ionophores could be associated with bacterial resistance against antimicrobials used in human medicine, and that resistant bacteria could spread to humans both by direct contact with animals and through food supply [2,9]. These considerations have led to increased focus on conventional broilers raised without the use of any in-feed antimicrobial agents, including AGPs as well as ionophores and other coccidiostats. In 2015/2016, the Norwegian broiler industry abolished the routine use of in-feed coccidiostats, including narasin [10].

The former widespread practice of supplementing broiler feeds with AGPs was mainly based on the favorable influence of these compounds on production performance [2]. Impaired production performance leading to increased production costs is a main concern associated with rearing broilers without in-feed antimicrobials. The traditionally most commonly used AGPs are predominantly active against gram-positive bacteria [11], and many of these antimicrobials have been shown to suppress the proliferation of CP in vivo [12,13] and in vitro [14,15,16]. Several studies report an association between increased numbers of intestinal CP and growth depression in chickens [12,17,18], and collectively these findings suggest that antibacterial activity against CP may be involved in the ‘antibiotic growth effect’. Development of NE and a subclinical form of this disease is associated with impaired production performance, cholangiohepatitis and high numbers of intestinal and fecal CP [19,20,21]. Infection with *Eimeria* spp. is considered an important predisposing factor for CP proliferation and development of NE in chickens [22,23].

The interest in non-antibiotic feed additives (hereafter: alternatives to antibiotics, ATAs) that might facilitate the abolishment of continuous use of in-feed AGPs and coccidiostats has increased during the recent years. Numerous new feed additives have reached the global poultry feed market. Different ATAs, including products based on probiotics, prebiotics, phytogenics and/or organic acids, claim to exert beneficial effects related to productivity, intestinal functions and intestinal health in broiler chickens.

Probiotics are based on non-pathogenic and non-toxigenic live microorganisms (e.g., bacteria or yeasts) supposed to provide health benefits to the host. Possible modes of action of probiotics include colonization of the intestine, competitive exclusion of other microorganisms, production of specific metabolites and stimulation of the immune system [24]. Two categories of probiotics are non-spore forming bacteria (e.g., *Lactobacillus* spp., *Enterococcus* spp., *Bifidobacterium* spp.) and bacterial spore formers (e.g., *Bacillus* spp.) [25]. Regulatory agencies have been reluctant to approve undefined microbial products due to the uncertainty of a consistent composition of the products. This concern has paved the way for defined probiotic products based on one or a few known strains.

Prebiotics are non-digestible feed ingredients assumed to stimulate proliferation and/or activity of intestinal microorganisms, which leads to beneficial physiological responses in the host [26]. Intake of prebiotics may increase the number of specific microbes and change the composition of the intestinal microbiota [27]. Examples of prebiotic compounds are complex carbohydrates derived from plants or yeasts, such as fructooligosaccharides (FOS), mannanoligosaccharides (MOS) and β-glucans [28,29]. In addition to selective promotion of beneficial bacteria, suggested modes of action of prebiotics are blocking pathogen adhesion, altering gene expression, affecting gut morphological structure and immunomodulation [29].

Phytogenic feed additives are based on bioactive compounds derived from plants, and a multitude of such plant products can broadly be classified as herbs or spices [28]. Examples of biologically active components and substances from plants are essential oils, oleoresins, tannins, saponins, flavonoids, alkaloids and resin acids. Various functions among plant-based products have been suggested, including antimicrobial, antiviral, antioxidative, anti-inflammatory and flavoring effects [30]. The compositional variation is considerable due to biological factors such as plant species, growing conditions, climate, harvest and manufacturing processes, and it is thus challenging to identify and evaluate the functional basis of this broad group of active components [31].

Organic acids of various lengths and their corresponding salts or esters are widely used as feed additives in livestock production and can be used individually or as blends of multiple acids. They may vary considerably in functionality due to number of carbon atoms and may be aliphatic or aromatic. Many organic acids consist of carboxylic acids and are natural constituents of animal or plant tissue or products of microbial fermentation. Industrially produced organic acids often come as salts or esters and in a coated or encapsulated form [31]. Carboxylic acids with an aliphatic chain are designated fatty acids. The subgroup short-chain fatty acids (SCFAs, 1–5 carbon atoms; C1–C5) are aliphatic compounds produced in nature by microbial fermentation of carbohydrates in the hindgut of humans and animals. The subgroup medium-chain fatty acids (MCFAs, 6–12 carbon atoms; C6–C12) are aliphatic compounds formed in nature predominantly in plants and extra-intestinal animal tissues. Suggested effects of organic acids are antibacterial activity through pH-regulation and changes in microbiota composition, immunomodulatory action and stimulation of the gut mucosa [28,29,31]. The heterogeneity of this feed additive category makes it difficult to define common properties and function, and the effects of different organic acids may vary considerably. It has been proposed that SCFAs can act directly upon the cell wall of gram-negative bacteria, and that fatty acids with longer chains can incorporate themselves into the cell membrane of gram-positive bacteria and promote leakage [32].

A multitude of studies on the impact of alternative feed additives in broiler chickens have been published. However, most studies focus on only one or a few additives within one or two ATA classes. Furthermore, these studies often differ with regard to a number of factors that may influence the results (e.g., housing of chickens, number of replicates and challenge), which makes it difficult or impossible to compare results across studies. Another problematic issue is publication bias that occurs when only results that show significant findings are reported [33]. These considerations make it relevant to study the effect of ATAs under uniform testing conditions.

The present study was conducted in order to examine the effect of commercially available ATAs from four different product classes on production performance and cecal CP counts. Feed additives were selected on the basis of being marketed with claimed beneficial effects on production performance, intestinal function and/or intestinal health in poultry. Production performance was recorded during two separate age levels; days 0–14 and days 14–28. CP counts were recorded during the fourth week of rearing, four to six days after challenge with *Eimeria* spp.

The aims of the study were to (a) evaluate the performance of the collective ATA group, (b) compare effects of classes of ATAs (probiotics/prebiotics/phytogenics/organic acids) and (c) identify active components or component combinations with beneficial effects on production performance and CP counts, with emphasis on the time span following *Eimeria* challenge.

## 2. Materials and Methods

### 2.1. Animals and Housing

Six trials were carried out at Scandinavian Poultry Research in Våler, Hedmark, Norway, using one-day-old Ross 308 broiler chickens obtained from a commercial hatchery (Nortura Samvirkekylling, Våler, Norway). The chickens were housed in floor pens of 5.6 m^2^ on new wood shavings in a climate-controlled poultry research facility, with a 50/50 female-to-male ratio per pen. Water and pelleted feed were given *ad libitum*. The chickens were exposed to light for 23 h a day on the first two days. For the rest of the experimental period, the chickens were exposed to light during 2 × 8 h a day, interrupted by 4 h periods of darkness. Apart from a 10-fold dose of Paracox-5 vet. on day 17 or 18, no vaccines were administered throughout the study. The study period lasted from day of hatch until day 28. Animal experiments were approved by the national animal research authority (Norwegian Food Safety Authority, approval ID 8179), and performed in accordance with national and international guidelines for the care and use of experimental animals.

### 2.2. Experimental Design

In each of the six trials, a total of 5280 one-day-old Ross 308 broiler chickens were randomly allocated into six experimental groups, each group comprising 11 replicate pens with 80 chickens per pen. All trials had similar design, and included four treatment groups receiving feed with a specific ATA product or a combination of two ATA products, a positive control group (NAR) receiving feed with the polyether ionophore and coccidiostat narasin (Monteban, Elanco Animal Health, Greenfield, IN, USA), and a negative control group (NEG) receiving feed with neither antimicrobial feed additives (AGPs or coccidiostats) nor ATA products. Feed additives were added to the feeds at an inclusion rate recommended by the manufacturers. No AGP products were included in this study, and narasin was used as a sole coccidiostat in the NAR group. The chickens were fed wheat-based starter and grower diets based on Ross Broiler Nutrition Specifications adapted to Norwegian broiler production from 0 to 14 and 14 to 28 days of age, respectively (Table 1).

In the five initial trials, 20 commercially available ATA products were evaluated individually for their effect on production performance and cecal CP counts. In the sixth trial, combinations of two ATA products per treatment group were evaluated using the same outcome variables. Products included in the sixth experiment were selected for testing due to promising impact on either production performance or CP counts in the five initial experiments. Products with positive effects on production performance were combined with products with CP reducing effect in order to study potential synergy effects. Descriptions of active ingredients and dose levels of the feed additives and feed additive combinations tested are listed in Table 2. Composition of the products and dosage levels are based on information given by the feed additive manufacturers on their web sites or as a response to our request.

On day 17 (one trial) or 18 (five trials) post hatch, all treatment groups in all six trials were challenged with a 10-fold dose of Paracox-5 vet. (MSD Animal Health, Boxmeer, the Netherlands) containing live, sporulated oocysts from five attenuated strains of *Eimeria* spp. (one precocious line each of *Eimeria acervulina* [approximately 5750 oocysts per broiler], *Eimeria mitis* [approximately 11,500 oocysts], and *Eimeria tenella* [approximately 5750 oocysts], and two precocious lines of *Eimeria maxima* [approximately 3450 oocysts]) in the drinking water.

### 2.3. Clostridium Perfringens Quantification

On days 4, 5 and 6 after *Eimeria* challenge, 11 chickens per treatment group (1 chicken from each replicate pen) were randomly selected and humanely euthanized by cranial stunning immediately followed by cervical dislocation before necropsy. Samples of cecal contents were collected in sterile stomacher bags and directly subjected to cultivation in order to quantify CP. In brief, the samples were diluted 1:100 in peptone saline water (0.1% peptone, Difco Laboratories Inc., Detroit, US and 0.85% NaCl) and homogenized for 30 s in a stomacher (Bagmixer 400 CC, Interscience, Saint Nom, France). Serial dilutions were made with non-buffered peptone water until a dilution of 10^–6^ was reached. Aliquots of 100 µL from the dilutions 10^–2^, 10^–4^ and 10^–6^ were plated onto sheep blood agar plates (Oxoid Blood Agar Base No.2 and 5% sheep blood, manufactured by the Norwegian Veterinary Institute, Oslo, Norway). The plates were incubated anaerobically at 37 °C for 24 h (Genbox anaer, Biomérieux, Marcy-l’Étoile, France). Single colonies with double hemolysis were counted, and colony-forming units per gram (cfu/g) cecal contents were calculated based on the given dilution. Typical colonies were selected for pure cultivation and later confirmed as CP by a matrix-assisted laser desorption ionization time-of-flight (MALDI-TOF) mass spectrometer (Bruker Daltonics, Bruker Corp., Billerica, MA, USA).

### 2.4. Post Mortem Examination

The small intestine of all chickens that were sampled for CP quantification was opened longitudinally and examined for pathological changes indicating NE, and scored as follows (modified from [34]): necrotic enteritis negative with no macroscopic mucosal ulcers or pseudomembranes, or necrotic enteritis positive with minimum one mucosal ulcer or pseudomembrane.

### 2.5. Production Performance Measurements

The amount of feed per pen was weighed when allocated and remaining feed was weighed before being discarded at feed change and at the end of the experiment. Accumulated feed intake per pen from days 0 to 14, 14 to 28 and 0 to 28 was calculated. Total live chicken weights per pen were recorded on days 0, 14 and 28, and mean body weight gain (BWG, g/chicken) and mean feed conversion ratio (FCR, g feed intake/g weight gain) per pen were calculated.

### 2.6. Statistical Analysis

Data on production performance and CP counts were examined on three different levels; (a) the impact of ATAs as one collective group (group level), (b) the impact of classes of ATAs (class level), and (c) the impact of individual ATA treatments (treatment level). On all levels, ATAs and the positive control with narasin-supplemented feed (NAR) were compared against the negative control with no feed additive (NEG). Frequencies of broilers with NE lesions were analyzed only on group level using Pearson’s chi-squared test in Stata version 14.2 (StataCorp LLC, College Station, TX, USA). Production performance and CP count data were analyzed using regression analyses in R version 3.5.3 (R Foundation for Statistical Computing, Vienna, Austria).

Production performance data were analyzed with pen as the unit of concern. Body weight gain and feed conversion ratio was obtained in the periods 0–14 days, 14–28 days and 0–28 days for groups, classes and treatments tested in six trials. The outcome from the six different trials could not be compared directly due to intertrial variability. In order to validly compare results from six different trials, it was necessary to control for the effect of trial in the statistical analysis. The principle approach to achieve such control was to use the results from NEG in each of the six trials as indicators of trial effect. A mixed-effects model (1) with only intercept (a) was used to obtain a trial-specific random effect ($\varepsilon_{Trial})$ for each outcome variable ($y_{Neg}$) per trial based on results from NEG using the package *lme4* in R [35].

(1) $$y_{Neg}=\;a+\varepsilon_{Trial}$$

For each of the outcome variables (y), results achieved in the different trials were adjusted with a value equal to the random effect obtained for the respective trial. Results across trials were compared using regression analysis (2) with ATA group/class/treatment (x) as fixed-effect variable and trial-specific random-effects from NEG as offset variable ($\varepsilon_{Trial}$). b represents the estimated parameters in the model.

(2) $$y=\;\varepsilon_{Trial}+b\cdot x\;$$

The necessity of adjustment for trial effect was calculated by the intraclass correlation coefficient (ICC), which is variance explained by the random effect divided by total variance of the residuals for the model based on all observations from NEG. Extreme outlier pens that were highly influential on the estimated regression results were identified using the function *outlierTest* from the package *car* in R [36]. Residuals from the regression models were visually inspected using the functions *qqnorm* and *qqline* in R and found to follow a normal distribution. The production performance results were reported in tables as means with standard deviation. Differences from NEG with *p* < 0.05 were accepted as statistically significant differences.

CP counts in cecal samples were analyzed with individual chicken samples as unit of concern. Since the residuals from the regression model did not follow a normal distribution, the CP count numbers were log transformed in order to fulfil this requirement. The effect of trial was controlled by adjusting for obtained random effect as described above, and subsequently regression analysis with ATA group/class/treatment as fixed-effect variable and trial-specific random-effects from NEG as offset variable was conducted. The results were reported in tables as mean log_10_ colony forming units per gram cecal content. Estimated mean log_10_ CP counts with 95% confidence interval for each treatment were presented in a graph where feed additive classes are indicated with different colors.

## 3. Results

### 3.1. Impact of the Collective ATA Group on Necrotic Enteritis, Intestinal CP Counts and Production Performance

Broilers with necrotic enteritis lesions during days 4–6 after *Eimeria* challenge constituted 8.1% among chickens from the NEG group (no feed additive, *n* = 198 chickens), 4.4% in the collective ATA group (24 ATA treatments, *n* = 792 chickens) and 0.5% in the NAR group (in-feed narasin, *n* = 198 chickens). Statistical analyses indicated significant difference in NE occurrence between the NEG group and the ATA group (*p* < 0.05), and between the ATA group and the NAR group (*p* < 0.01).

The ATA group reduced CP counts in intestinal contents from log_10_ 6.09 to log_10_ 5.63 cfu/g (*p* = 0.005), corresponding to a 65% reduction in non-transformed counts (Table 3). This substantial reduction was, however, moderate as compared to the very strong effect of narasin (from log_10_ 6.09 to log_10_ 2.92 cfu/g (*p* < 0.001), corresponding to a 99.9% reduction in non-transformed counts).

Both the ATA group and the NAR group had strongest beneficial impact on production performance during days 14–28, i.e., the age interval characterized by intestinal stress induced by *Eimeria* challenge on day 17 or 18. The collective ATA group demonstrated a 1.6% improvement (*p* < 0.001) in FCR during days 14 to 28 (FCR_14–28_) and a 2.8% increase (*p* < 0.001) in BWG during days 14 to 28 (BWG_14–28_) compared to the NEG group (Table 3). The beneficial effect of the ATA group on production performance was not as pronounced as the positive effect of narasin (4.9% improved FCR_14–28_ and 7.8% increased BWG_14–28_).

### 3.2. Impact of ATA Classes on Intestinal CP Counts and Production Performance

Four ATA classes (probiotics, PRO; prebiotics, PRE; phytogenics, PFA; organic acids, OA), a set of treatments each based on more than one ATA class (mixed products, MIX) and NAR (i.e., narasin) were compared with NEG (i.e., no feed additive) (Table 4). Although all ATA classes demonstrated a reducing effect on numbers of CP per gram intestinal contents, only two classes (PFA and PRO) showed statistically significant reduction (*p* < 0.05). The estimated reducing impacts of PFA and PRO were 87% and 75% in non-transformed CP counts, respectively, when compared to NEG.

Three ATA classes (PRO, PRE and MIX) improved FCR_14–28_ (1.3%–2.7% improvement, *p* < 0.01), and four classes (PRO, PRE, OA and MIX) increased BWG_14–28_ (2.8%–3.9% increase, *p* < 0.01). Accumulated feed conversion during days 0 to 28 (FCR_0–28_) was improved by all ATA classes (1.1%–1.5%, *p* < 0.01). However, only the OA class improved feed conversion during days 0 to 14 (FCR_0–14_) significantly (3.3%, *p* < 0.001). Narasin outperformed the ATA classes at all age intervals, except for body weight gain during days 0 to 14 (BWG_0–14_) and FCR_0–14_, where the OA class performed similarly.

### 3.3. Impact of Treatments on Intestinal CP Counts and Production Performance

**Figure 1:**
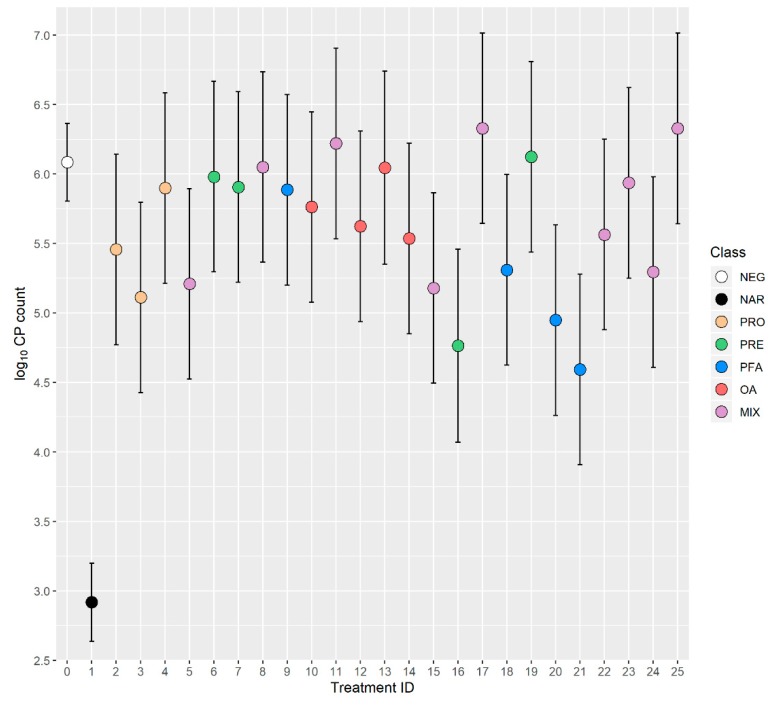
Cecal *Clostridium perfringens* (CP) counts with 95% confidence intervals. Negative control (NEG) is treatment 0, narasin (NAR) is treatment 1, probiotics (PRO) are treatments 2–4, prebiotics (PRE) are treatments 6, 7, 16 and 19, phytogenics (PFA) are treatments 9, 18, 20 and 21, organic acids (OA) are treatments 10, 12, 13 and 14, and mixed products (MIX) are treatments 5, 8, 11, 15, 17 and 22–25.

Intestinal CP counts were significantly reduced (*p* < 0.05) by 8 out of 24 ATA treatments (ID 3, 5, 15, 16, 18, 20, 21 and 24) as shown in Table 5. Estimated reduction in non-transformed CP counts among these eight treatments ranged from 84% to 97% when compared to NEG. Phytogenic components were present in 5/8 treatments (ID 15, 18, 20, 21 and 24), prebiotic components in 3/8 treatments (ID 5, 16 and 24), probiotic components in 2/8 treatments (ID 3 and 5) and OA components were present in 2/8 treatments (ID 15 and 24). Mean log_10_ CP counts with 95% confidence interval for each ATA treatment are shown in Figure 1.

FCR_14–28_ was improved (*p* < 0.05) by 10/24 tested ATA treatments (Table 5). Five of these treatments (ID 4, 6, 7, 8 and 9) achieved FCR_14–28_ improvements (3.2% to 5.2%, *p* < 0.001) that returned the same significance level as narasin (4.9% improvement, *p* < 0.001). These five treatments had active components classified as probiotics (ID 4), prebiotics (ID 6 and 7), phytogenics (ID 8 and 9) or organic acids (ID 8). In total, 13/24 ATA treatments improved FCR_0–28_ (1.4% to 3.2% improvement, *p* < 0.05). Seven of these treatments (ID 6, 7, 8, 9, 11, 13 and 24) achieved improvements in FCR _0–28_ that returned the same significance level (2.1% to 3.2% improvement, *p* < 0.001) as narasin (4.5% improvement, *p* < 0.001).

BWG_14–28_ and body weight gain during days 0 to 28 (BWG_0–28_) were increased by 10/24 and 8/24 ATA treatments, respectively. Two treatments (ID 11 and 13) excelled in increasing both these parameters, with a significance level similar to narasin (*p* < 0.001).

In the sixth trial, two-product combinations of treatments with predominantly CP-reducing impact (ID 5, 16 and 21) and treatments with predominantly production performance-promoting impact (ID 7, 11 and 13) were evaluated (comprising treatment ID 22–25 in Table 2). Treatment 16 did not appear to reduce the FCR-improving effect of treatments 11 and 13 (Table 5) but tended to diminish the growth promoting impact of these treatments. Treatment 5 seemed to diminish the FCR-improving effect and remove the growth-promoting effect of treatment 7. Treatment 21 appeared to reduce or remove the improvement in FCR and to remove the growth-promoting effect of treatment 7. On the other hand, treatment 7 seemed to remove the CP-reducing impact of treatments 5 and 21, and treatment 13 appeared to remove the CP-reducing impact of treatment 16. In contrast to these results, treatment 11 did not appear to impair the CP-reducing impact of treatment 16. As a result of these interactions between predominantly CP-reducing and production performance-promoting single treatments, treatment 24 was the only one of four tested product combinations (ID 22–25, Table 5) with beneficial effects on production performance variables as well as CP counts.

### 3.4. Active Components with Combined Beneficial Effects on FCR_14–28_ and CP Counts

In total, 10/24 and 8/24 tested treatments improved (*p* < 0.05) FCR_14–28_ and CP counts, respectively. Collectively these treatments comprised a group of 16 treatments; 14 treatments either improved FCR_14–28_ or reduced CP counts, and only two treatments (ID 3 and 24) influenced both FCR_14–28_ and CP counts in a beneficial way. One of the two superior treatments according to these criteria was a probiotic (ID 3) with the *Bacillus subtilis* PB6 strain as the only active component. The other treatment (ID 24) was a combination of three ATA classes (OA/PFA/PRE) and two products; one product (ID 16) containing whole cells of *Saccharomyces cerevisiae* and its metabolites, and another (ID 11) being a mixture of SCFAs (including C4), MCFAs (including C12) and a phenolic compound.

## 4. Discussion

The collective group of 24 ATA treatments tested in this study reduced the occurrence of NE and reduced intestinal CP counts after *Eimeria* challenge. Production performance measured as BWG and FCR was improved by the collective ATA group, but not significantly during the phase prior to *Eimeria* challenge. These results indicate a beneficial effect of several ATAs when chickens are exposed to mild to moderate intestinal stress. The favorable effects of the polyether ionophore narasin on CP counts and production performance were numerically and in part significantly stronger than the effect of the collective group of ATA treatments.

In this study, the chickens were challenged orally with five precocious lines of *Eimeria* spp. as a predisposing factor for NE. NE is expected to appear during oocyst excretion, which begins between three and four days following inoculation with precocious *Eimeria acervulina* [37] and *Eimeria mitis* lines, and presumably later with precocious *Eimeria maxima* and *Eimeria tenella* lines [38]. In a previous study with a similar type of *Eimeria* challenge, most gut damage was detected four to six days after inoculation [39]. Postmortem examinations in this study confirmed the presence of NE during this time span after *Eimeria* challenge. Intestinal CP counts are strongly associated with NE [19,34,40]. Furthermore, increased occurrence of NE in commercial broiler flocks has been associated with impaired accumulated FCR at slaughter [20]. Weakened production performance is likely to be most pronounced during the part of the rearing phase that is affected by NE. Based on the considerations mentioned above we chose to emphasize CP counts on days four to six after *Eimeria* challenge and FCR_14–28_ in our analyses of effect of ATAs on intestinal health.

The evaluation of ATAs can rest on different criteria, depending on point of view, practical circumstances and current health problems. Disease conditions associated with increased metabolism and rapid growth in broilers (in particular cardiovascular and musculoskeletal disorders) are generally important, and it has been claimed that such conditions cause greater economic loss than infectious agents [41]. In a recent study, it was found that chickens with higher body weight and BWG were predisposed to develop more severe NE lesions when challenged with CP [42]. Although attractive in the short run, increased weight gain may therefore come at a cost not only to chicken health and welfare but also to the farmers’ economy and a sustainable use of feed resources. In light of this consideration, we have emphasized the effect on FCR as production performance parameter, because it is an indicator of intestinal health as well as resource efficiency.

The only two ATA treatments (ID 3 and 24) with a combined beneficial effect on CP counts (84 to 89% reduction, *p* < 0.05) and FCR_14–28_ (2.3% improvement, *p* < 0.05) were based on different types of active components. One of the treatments (ID 3) was a mono-strain (*Bacillus subtilis* strain PB6) spore-forming bacterial probiotic. This probiotic strain has been reported to inhibit CP in vitro [43] and improve FCR [44], which is in agreement with our findings.

The other treatment (ID 24) was based on a heterogeneous collection of active components including short- and medium-chain fatty acids, a phenolic compound and dehydrated whole cells and metabolites of the yeast *Saccharomyces cerevisiae* (SC). This treatment comprised two commercial products that were also tested individually (ID 11 and 16). Whereas the yeast product (ID 16) alone demonstrated a 95% reduction (*p* < 0.001) in non-transformed CP counts, the product containing a blend of organic acids and a phenolic compound (ID 11) had no reducing impact on CP. Viewed against this background it seems probable that the CP reducing effect of treatment 24 was mainly associated with one or several yeast components found in treatment 16. In addition to treatment 24, three other ATA treatments based on the yeast SC were tested. These treatments, which were based on SC cell wall extracts (ID 6, 7 and 19), did not reduce CP counts to an extent that was significant with the sample size and/or feed additive dosage used in our study, whilst the treatments based on SC whole cells and metabolites (ID 16 and 24) did. No previous reports on the effect of SC metabolites and SC whole cells on CP counts in broilers have been found. Regarding yeast cell wall extracts, previously published literature has indicated both significant [45] and non-significant [46] CP-reducing impact. Our results indicate that whole cells and/or metabolites of SC inhibited intestinal CP growth more efficiently than SC cell wall extracts with the product inclusion levels used in this study.

One of the active components in treatment 11 was lauric acid (C12), a MCFA that has been demonstrated to inhibit CP in vitro [47]. Treatment 11 did, as mentioned above, not reduce CP counts when used as sole feed additive in this study. Possible explanations include too low concentration of lauric acid and/or interfering effects by other treatment components.

Regarding production performance, the combination of treatments 11 and 16 (i.e., treatment 24) had a significantly beneficial effect on FCR_14–28_. However, neither of these two treatments improved FCR_14–28_ when tested individually. This finding suggests a synergy effect with regard to FCR_14–28_ between active components present in the two products. Beneficial effects of dietary supplementation of whole cells and metabolites of SC on production performance in broilers have been reported by others [48].

The combination of SCFAs (including butyric acid-C4), MCFAs (including lauric acid-C12) and a phenolic compound in treatment 11 generated the numerically highest weight gain (BWG_0–14_ and BWG_14–28_) of all ATA treatments in this study but had no apparent impact on CP counts. This result suggests that rapid growth is possible in the presence of relatively high cecal CP counts. A possible explanation could be that this treatment reduced the counts of virulent CP strains (e.g., strains harbouring the *netB* gene) or the expression of virulence factors (e.g., the NetB toxin), but not the total CP counts. Treatment 11 might also have influenced the intestinal microbiota in a way that neutralized the negative impact of high CP counts.

Six of 24 ATA treatments (ID 5, 15, 16, 18, 20 and 21) were associated with reduced CP counts (at least 83% reduction in non-transformed counts, *p* < 0.05) without improving FCR_14–28_ significantly. Treatment 21 had a very strong reducing impact on CP counts (a 97% reduction, *p* < 0.001) and improved FCR_0–28_ (1.8%, *p* < 0.01), but had only a numerically (1.3%, non-significant) beneficial impact on FCR_14–28_. Active components of treatment 21 included oleoresins from turmeric (*Curcuma longa*) and chili peppers (genus *Capsicum*). These results are in agreement with reports on inhibitory activity against CP of turmeric extracts [49], reduced gut lesion scores in CP-challenged broilers treated with *Capsicum* and *Curcuma* oleoresins [50,51] and improved cumulative FCR of turmeric powder [52]. Treatment 20 was based on tall oil fatty acids from coniferous trees including resin acids. Resin acids have been reported to inhibit CP in vitro [53], and our results suggest similar effects in vivo.

Treatments 22–25 were tested in a final trial intended to evaluate two-product combinations of treatments improving production performance and treatments with CP-reducing impact. Treatment 24 was the only combination with beneficial impact on both CP counts and production results. These results suggest that the interaction between predominantly CP-reducing and production-promoting components vary substantially. Among three tested CP-reducing treatments in the final trial (ID 5, 16 and 21), a treatment based on dehydrated SC culture with whole cells and metabolites (ID 16) was the least impairing with regard to the production-promoting effects of its combination treatment. Among the three tested production performance-improving treatments (ID 7, 11 and 13), treatment 11 based on short- and medium-chain fatty acids and other components was the only one that did not impair the CP-reducing impact of its combination treatment. More work is needed to identify the role of the different components in treatments 11 and 16, and whether the beneficial interaction of these components also can be extended to include other CP-reducing and production-promoting components.

Our findings indicate that a reduction of CP counts induced by ATAs was not always associated with improved production performance. Lack of a positive impact on feed efficiency and growth rate has also been documented with regard to ionophores under certain conditions [54], in spite of these compounds’ suppressing effect on CP counts. However, when used at recommended concentrations in broiler flocks exposed to coccidia, the net effect of ionophores is usually improved performance. In our study, considerably improved performance combined with a strong CP-reducing effect of the ionophore narasin was present. These results confirm that our challenge model worked as expected, and that the in-feed concentration of narasin was within the optimal range. The reason why some of the ATAs with CP-reducing effect in our study did not induce a significantly positive net impact on production performance under the same test conditions remains unclear. Possible explanations may be that the inhibiting effect on CP was accompanied by reduced ability to utilize feed efficiently and/or establishment of another performance-impairing intestinal microbiota.

Eight of 24 ATA treatments (ID 2, 4, 6, 7, 8, 9, 13 and 25) improved FCR_14–28_ (at least 2.0% improvement, *p* < 0.05) without reducing CP counts significantly. One of these treatments (ID 4) was a mono-strain *Bacillus subtilis* probiotic. Data from other studies demonstrate the capacity of *Bacillus subtilis* strains to suppress the growth of CP and improve production performance and intestinal morphology [55,56,57]. However, the favorable impact on FCR_14–28_ in this study might have been caused by other mechanisms than inhibition of CP growth. Suggested modes of action associated with probiotics are maintenance of balanced microbial populations, modulation of the host immune system, promotion of epithelial barrier integrity and alteration of villus length and crypt depth [44,58,59,60,61].

Two (ID 6 and 7) of the treatments improving FCR_14–28_ but not CP counts contained cell wall extracts from the yeast SC. Both treatments 6 and 7 had a considerably beneficial impact on FCR_14–28_ (estimated 4.3 and 5.2% reduction, respectively). Of the SC cell wall-based treatments, the products with the apparently highest content of β-glucans (ID 6 and 7) had the best effect on FCR_14–28_ as compared with the other yeast cell wall-based treatment in our study (ID 19). Treatment 7, containing minimum 60% purified β-1.3/1.6 glucans, even outperformed narasin numerically with regard to FCR_14–28_. These findings suggest that SC-derived β-glucans are potent when it comes to improvement of FCR in broilers exposed to *Eimeria* spp. Beneficial effects of yeast β-glucans on performance in broilers are supported by some [62,63] and in contradiction with results from other previous reports [64,65]. Possible explanations for the FCR_14–28_-promoting effect of feed additives containing β-glucans are modulations of the immune response [62,64].

Two other treatments (ID 8 and 9) with favorable effect on FCR_14–28_ without significant reduction of CP counts contained essential plant oils. Essential oil components in treatments 8 and 9 included thymol (in both treatments), eugenol and piperine (in treatment 8) and carvacrol, anethol and limonene (in treatment 9). In treatment 8, essential oils were combined with benzoic acid. Published results on effects and mode of action of essential oils suggest that several of these compounds inhibit the growth of CP [66,67,68], although the findings are not always clear cut [69], or they show no effect on CP counts [70]. Reports on mitigation of gut lesions in chickens challenged with CP [67,71] underpin the view that at least thymol and carvacrol suppress the pathogenic action of CP. Studies on the effect of essential oil components on production performance reveal variable results. One study reports a negative effect on broiler performance using a blend of thymol, eugenol, curcumin and piperine [70], another describe a non-significant tendency of improved FCR_14–28_ using a blend of carvacrol and thymol [71], and a third study presents significant improvement of FCR_0–28_ by carvacrol but not by thymol [72]. The lack of standardization of studies, including variable feed additive dosage and different combinations of active compounds, makes comparison of results from different studies difficult. The interpretation of results is further complicated by the multiple suggested effects of different essential oils, including antibacterial and antioxidant properties, enhancement of the immune system, and stimulation of digestive secretions and blood circulation [73,74]. Regardless of mechanism, these two predominantly phytogenic feed additives (ID 8 and 9) had a pronounced beneficial effect on FCR_14–28_ on par with narasin in the current study.

The apparent lack of a CP-reducing effect of yeast cell wall extracts, essential oils and other active components associated with improved FCR_14–28_ in this study may in part be related to experimental design. As observed from our results (Table 5), estimated CP count reductions of 76% or less (e.g., treatment ID 2 and 14) returned non-significant (>0.05) *p*-values. The main reason for this low statistical power was high variance of CP counts in individual observations within each treatment, leading to imprecise estimates. Our experiments were designed with 33 replicates of individual CP counts per ATA treatment, and this sample size returned relatively wide confidence intervals (as shown in Figure 1). The statistical analysis involving the whole ATA group (Table 3) indicated that when 792 individual samples with a log_10_ 5.63 CP estimate were compared with the NEG group with 198 individual samples and a log_10_ 6.09 estimate (corresponding to a 65% difference), this difference was significant (*p* = 0.005).

The ATAs did not suppress CP counts to the same extent as the ionophorous coccidiostat narasin. The superior results of narasin in this respect were most likely due to the strong antibacterial effect of this compound. Narasin has been reported with inhibitory effect on CP growth similar to or better than antibiotics used as drinking water medication for poultry [75].

Different ATAs can add value to the broiler chicken industry in several ways. Some improve BWG and/or FCR, others inhibit growth of CP or have a beneficial effect on both production performance parameters and intestinal CP counts. The use of specific ATAs could possibly be targeted to specific age intervals or current health status in the flock. Future studies of the impact of ATAs on intestinal CP counts would most likely benefit from modified sampling protocols and quantification methods. Study designs that were useful for investigating the effect of AGPs and ionophorous coccidiostats should not be copied without reservation when studying non-antibiotic alternative feed additives. Finally, a less pronounced effect than narasin of selected ATAs on production performance and/or CP counts in this study does not necessarily mean that the impact is of no importance to broiler health and production economy.

In this study, ATA classes displayed distinct performance profiles. The probiotic class reduced CP counts and improved production performance during the time period with intestinal stress (days 14–28), but impaired weight gain during days 0–14. The prebiotic class improved production performance during days 14–28 and had a non-significantly reducing impact on CP counts. The phytogenic class had a markedly reducing impact on CP counts and improved FCR_0–28_. The organic acid class increased weight gain throughout the study period and improved FCR_0–14_ but did not reduce CP counts significantly. These findings suggest that employing ATA classes for specific purposes may be useful. As an example, combining probiotic and organic acid treatments might boost production performance throughout the grow-out period and at the same time reduce CP counts during intestinal stress. In this study, we tested other ATA class combinations with variable results, indicating the need for testing of specific combinations of active components within the ATA classes.

## Figures and Tables

**Table 1:** Diet composition ^1^.

Chemical Composition	Starter Diet ^2^	Grower Diet ^3^
(g/kg feed)	0–14 days	14–28 days
Dry matter	887.2	881.3
Crude protein	239.6	222.0
Crude fat	67.8	99.6
Crude fiber	30.3	29.0
Nitrogen-free extracts	493.7	479.0
Ash	55.8	51.7
Lysine	14.0	12.9
Methionine + Cysteine	11.6	11.1
Threonine	9.4	9.0
Tryptophan	2.7	2.5
Arginine	13.8	12.7
Calcium (Ca)	9.2	7.4
Phosphorus (P)	6.3	5.9
Sodium (Na)	1.4	1.6
Potassium (K)	7.7	7.4
Chloride (Cl)	2.3	2.2
Magnesium (Mg)	1.6	1.6
NSP enzymes ^4^ and phytase	0.15	0.15
Metabolizable energy (MJ/kg)	12.13	12.78

^1^ Mean values from diets in six trials. ^2^ Vitamins and minerals: Cu 15 mg/kg; Zn 82 mg/kg; Mn 126 mg/kg; Se 0.27 mg/kg; I 1.04 mg/kg; Fe 52 mg/kg; Vit.A 9575 IU; Vit.E 96 IU; Vit.D3 4994 IU; Vit.K 7.0 mg/kg; Vit.B1 4.2 mg/kg; Vit.B2 7.3 mg/kg; Vit.B3 59.7 mg/kg; Vit.B5 20.0 mg/kg; Vit.B6 12.0 mg/kg; Vit.B12 0.02 mg/kg; biotin 2.1 mg/kg; folic acid 2.9 mg/kg; choline chloride 1726 mg/kg. ^3^ Vitamins and minerals: Cu 15 mg/kg; Zn 82 mg/kg; Mn 128 mg/kg; Se 0.27 mg/kg; I 1.05 mg/kg; Fe 53 mg/kg; Vit.A 9488 IU; Vit.E 81 IU; Vit.D3 4983 IU; Vit.K 5.6 mg/kg; Vit.B1 3.6 mg/kg; Vit.B2 6.8 mg/kg; Vit.B3 54.0 mg/kg; Vit.B5 18.0 mg/kg; Vit.B6 11.0 mg/kg; Vit.B12 0.02 mg/kg; biotin 2.4 mg/kg; folic acid 2.7 mg/kg; choline chloride 1500 mg/kg. ^4^ Non-starch polysaccharide enzymes.

**Table 2:** Treatment ID, class of feed additives, active components and inclusion rate of feed additive products.

ID ^1^	Class ^2^	Active Components and Product Description ^3^	Dosage ^4^ (Starter/Grower)
0	NEG	None	–
1	NAR	Narasin (100 g narasin/kg additive)	700/700
2	PRO	*Lactobacillus farciminis* CNMA 67/4R strain (1 × 10^9^ cfu/gram additive)	500/500
3	PRO	*Bacillus subtilis* PB6 strain (2 × 10^8^ cfu/gram additive)	500/500
4	PRO	One *Bacillus subtilis* strain, material no. 671265 (1.6 × 10^9^ cfu/gram additive)	500/500
5	PRO/PRE	*Enterococcus faecium* DSM 16211 (jejunum isolate), *Bifidobacterium animalis* DSM 16284 (ileum isolate), *Lactobacillus salivarus* DSM 16351 (caeca isolate) with mix ratio 3:1:6 (total cfu/gram: 2 × 10^8^), plant-derived fructooligosaccharides from inulin	1000/1000
6	PRE	*Saccharomyces cerevisiae* cell wall extracts (including typ. 25% β-1,3/1,6 glucans and min. 24% mannanoligosaccharides)	1000/1000
7	PRE	*Saccharomyces cerevisiae* cell wall extracts (including min. 60% purified β-1,3/1,6 glucans)	250/250
8	OA/PFA	Benzoic acid (80%–83%) and a blend of essential oils (including thymol 1.0%–1.9%, eugenol 0.5%–1.0%, and piperine 0.05%–0.1%)	300/300
9	PFA	Essential oil blend (min. 31.9%, including carvacrol, thymol, anethol and limonene)	150/150
10	OA	Medium-chain fatty acids (C6, C8 and C10)	1600/1600
11	OA/PFA	Short- and medium chain fatty acids (including C4 and C12), phenolic compound and organic acids	1500/1500
12	OA	Tri- and diglycerides of butyric acid (C4)	1000/1000
13	OA	Diformate derived from C1 (57% Na-formate, 39% formic acid)	3000/3000
14	OA	Lactylates (C12 and C14 esterified with lactic acid)	750/750
15	OA/PFA	Short- and medium-chain fatty acids (including monoglycerides of C3, C4, C8 and C10) and essential oils (mainly cinnamon aldehyde)	3000/2500
16	PRE	Dehydrated *Saccharomyces cerevisiae* culture with whole cells, metabolites and medium nutrients	1250/620
17	OA/PFA	Glycerol-esterified short- and medium-chain fatty acids (including C3, C4, C8 and C10) and 6% phytogenics (including essential oils, saponins and bitter and pungent substances)	750/750
18	PFA	Phytogenics including alkaloids, saponins, thymol and glyco-components derived from Yucca plants	2000/1000
19	PRE	*Saccharomyces cerevisiae* cell wall extracts (primarily mannanoligosaccharides)	800/400
20	PFA	Tall oil fatty acids from coniferous trees, including resin acids (8%–9%)	1000/1000
21	PFA	Oleoresins from turmeric (*Curcuma longa*) (4.4%) and chili peppers (genus Capsicum) (4.4%)	100/100
22	PRO/PRE	Active components of ID 5	1000/1000
	+PRE	and ID 7	250/250
23	PRE	Active components of ID 7	250/250
	+PFA	and ID 21	100/100
24	OA/PFA	Active components of ID 11	1500/1000
	+PRE	and ID 16	1250/625
25	OA	Active components of ID 13	3000/2000
	+PRE	and ID 16	1250/625

^1^ Treatment ID number. ^2^ NEG = negative control, NAR = positive control, PRO = probiotics, PRE = prebiotics, PFA = phytogenics, and OA = organic acids. ^3^ Based on available information from the product manufacturers. ^4^ Amount added product given as grams/ton feed in starter and grower diets.

**Table 3:** Body weight gain, feed conversion ratio and *Clostridium perfringens* counts for negative control, narasin and alternatives to antibiotics ^1^.

Group	Days 0–14		Days 14–28		Days 0–28		CP Counts log_10_ cfu/g
	BWG g	FCR g/g	BWG g	FCR g/g	BWG g	FCR g/g	
NEG ^2^	474 ± 4	1.098 ± 0.006	1240 ± 9	1.338 ± 0.005	1714 ± 11	1.248 ± 0.003	6.09 ± 0.14
NAR ^3^	488 ± 6 *p* = 0.032	1.064 ± 0.008 *p* < 0.001	1337 ± 12 *p* < 0.001	1.273 ± 0.007 *p* < 0.001	1825 ± 16 *p* < 0.001	1.192 ± 0.005 *p* < 0.001	2.92 ± 0.20 *p* < 0.001
ATAs ^4^	478 ± 5 *p* = 0.419	1.087 ± 0.006 *p* = 0.079	1275 ± 10 *p* < 0.001	1.317 ± 0.006 *p* < 0.001	1753 ± 12 *p* = 0.002	1.232 ± 0.004 *p* < 0.001	5.63 ± 0.16 *p* = 0.005
ICC ^5^	0.35	0.61	0.43	0.35	0.42	0.28	0.08

^1^ Results are reported as means ± standard deviation. Body weight gain (BWG) in grams/chicken, feed conversion ratio (FCR) in grams feed intake/grams weight gain and *Clostridium perfringens* (CP) counts as log_10_ colony forming units/gram cecal content. ^2^ Negative control (no feed additive); production performance data based on *n* = 66 pens, and CP data based on *n =* 198 individual chicken samples. ^3^ Narasin; production performance data based on *n* = 66 pens, and CP data based on *n =* 198 individual chicken samples. ^4^ Alternatives to antibiotics treatments; production performance data based on *n* = 264 pens, and CP data based on *n =* 792 individual chicken samples. ^5^ Intraclass correlation coefficient.

**Table 4:** Body weight gain, feed conversion ratio and *Clostridium perfringens* counts for negative control, narasin and classes of alternatives to antibiotics ^1^.

Class	Days 0–14		Days 14–28		Days 0–28		CP Counts log_10_ cfu/g
	BWG g	FCR g/g	BWG g	FCR g/g	BWG g	FCR g/g	
NEG ^2^	474 ± 4	1.098 ± 0.006	1240 ± 9	1.338 ± 0.005	1714 ± 11	1.248 ± 0.003	6.09 ± 0.14
NAR ^3^	488 ± 6 *p* = 0.032	1.064 ± 0.008 *p* < 0.001	1337 ± 12 *p* < 0.001	1.273 ± 0.007 *p* < 0.001	1825 ± 16 *p* < 0.001	1.192 ± 0.005 *p* < 0.001	2.92 ± 0.20 *p* < 0.001
PRO ^4^	455 ± 8 *p* = 0.012	1.113 ± 0.009 *p* = 0.118	1283 ± 15 *p* = 0.004	1.302 ± 0.009 *p* < 0.001	1736 ± 19 *p* = 0.239	1.232 ± 0.006 *p* = 0.004	5.49 ± 0.25 *p* = 0.017
PRE ^5^	479 ± 7 *p* = 0.496	1.095 ± 0.009 *p* = 0.761	1288 ± 14 *p* < 0.001	1.305 ± 0.008 *p* < 0.001	1767 ± 18 *p* = 0.003	1.229 ± 0.005 *p* < 0.001	5.70 ± 0.23 *p* = 0.092
PFA ^5^	480 ± 7 *p* = 0.375	1.086 ± 0.009 *p* = 0.152	1247 ± 14 *p* = 0.610	1.323 ± 0.008 *p* = 0.062	1727 ± 17 *p* = 0.457	1.233 ± 0.005 *p* = 0.004	5.18 ± 0.23 *p* < 0.001
OA ^5^	490 ± 7 *p* = 0.025	1.062 ± 0.009 *p* < 0.001	1288 ± 14 *p* < 0.001	1.325 ± 0.008 *p* = 0.114	1778 ± 17 *p* < 0.001	1.232 ± 0.005 *p* = 0.002	5.74 ± 0.23 *p* = 0.130
MIX ^6^	479 ± 6 *p* = 0.339	1.087 ± 0.007 *p* = 0.103	1275 ± 11 *p* = 0.002	1.320 ± 0.007 *p* = 0.007	1754 ± 14 *p* = 0.005	1.234 ± 0.004 *p* = 0.001	5.79 ± 0.19 *p* = 0.113
ICC ^7^	0.35	0.61	0.43	0.35	0.42	0.28	0.08

^1^ Results are reported as means ± standard deviation. Body weight gain (BWG) in grams/chicken, feed conversion ratio (FCR) in grams feed intake/grams weight gain and *Clostridium perfringens* (CP) counts as log_10_ colony forming units/gram cecal content. ^2^ Negative control (no feed additive); production performance data based on *n* = 66 pens, and CP data based on *n =* 198 individual chicken samples. ^3^ Narasin; production performance data based on *n* = 66 pens, and CP data based on *n =* 198 individual chicken samples. ^4^ Probiotics (PRO); production performance data based on *n* = 33 pens, and CP data based on *n =* 99 individual chicken samples. ^5^ Prebiotics (PRE), phytogenics (PFA), organic acids (OA); production performance data based on *n* = 44 pens, and CP data based on *n =* 132 individual chicken samples. ^6^ Mixed products (MIX), i.e., treatments based on more than one ATA class; production performance data based on *n* = 99 pens, and CP data based on *n =* 297 individual chicken samples.^7^ Intraclass correlation coefficient.

**Table 5:** Body weight gain, feed conversion ratio and *Clostridium perfringens* counts for negative control, narasin and alternatives to antibiotics treatments ^1^.

ID-Class	Days 0–14		Days 14–28		Days 0–28		CP Counts log_10_ cfu/g
	BWG g	FCR g/g	BWG g	FCR g/g	BWG g	FCR g/g	
0-NEG ^2^	474 ± 4	1.098 ± 0.006	1240 ± 9	1.338 ± 0.005	1714 ± 11	1.248 ± 0.003	6.09 ± 0.14
1-NAR ^3^	488 ± 6 *p* = 0.032	1.064 ± 0.008 *p* < 0.001	1337 ± 12 *p* < 0.001	1.273 ± 0.007 *p* < 0.001	1825 ± 16 *p* < 0.001	1.192 ± 0.005 *p* < 0.001	2.92 ± 0.20 *p* < 0.001
2-PRO ^4^	452 ± 11 *p* = 0.049	1.118 ± 0.014 *p* = 0.153	1285 ± 22 *p* = 0.044	1.305 ± 0.012 *p* = 0.007	1735 ± 29 *p* = 0.455	1.236 ± 0.008 *p* = 0.120	5.46 ± 0.38 *p* = 0.097
3-PRO ^4^	451 ± 11 *p* = 0.044	1.110 ± 0.014 *p* = 0.383	1273 ± 22 *p* = 0.132	1.307 ± 0.012 *p* = 0.012	1723 ± 29 *p* = 0.740	1.235 ± 0.008 *p* = 0.094	5.11 ± 0.38 *p* = 0.010
4-PRO ^4^	462 ± 11 *p* = 0.274	1.111 ± 0.014 *p* = 0.357	1290 ± 22 *p* = 0.024	1.295 ± 0.012 *p* < 0.001	1751 ± 29 *p* = 0.198	1.224 ± 0.008 *p* = 0.002	5.90 ± 0.38 *p* = 0.623
5-MIX ^4^	472 ± 11 *p* = 0.872	1.084 ± 0.014 *p* = 0.304	1268 ± 22 *p* = 0.207	1.329 ± 0.012 *p* = 0.459	1739 ± 29 *p* = 0.378	1.244 ± 0.008 *p* = 0.579	5.21 ± 0.38 *p* = 0.021
6-PRE ^4^	476 ± 11 *p* = 0.854	1.112 ± 0.014 *p* = 0.324	1305 ± 22 *p* = 0.004	1.280 ± 0.012 *p* < 0.001	1782 ± 29 *p* = 0.023	1.216 ± 0.008 *p* < 0.001	5.98 ± 0.38 *p* = 0.782
7-PRE ^4^	470 ± 11 *p* = 0.731	1.106 ± 0.014 *p* = 0.544	1311 ± 22 *p* = 0.002	1.269 ± 0.012 *p* < 0.001	1781 ± 29 *p* = 0.018	1.211 ± 0.008 *p* < 0.001	5.91 ± 0.38 *p* = 0.637
8-MIX ^4^	469 ± 11 *p* = 0.672	1.093 ± 0.014 *p* = 0.708	1293 ± 22 *p* = 0.016	1.280 ± 0.012 *p* < 0.001	1763 ± 29 *p* = 0.086	1.208 ± 0.008 *p* < 0.001	6.05 ± 0.38 *p* = 0.928
9-PFA ^4^	459 ± 11 *p* = 0.178	1.108 ± 0.014 *p* = 0.480	1288 ± 22 *p* = 0.030	1.284 ± 0.012 *p* < 0.001	1747 ± 29 *p* = 0.243	1.221 ± 0.008 *p* < 0.001	5.89 ± 0.38 *p* = 0.600
10-OA ^4^	499 ± 11 *p* = 0.029	1.073 ± 0.014 *p* = 0.070	1280 ± 22 *p* = 0.072	1.327 ± 0.012 *p* = 0.368	1780 ± 29 *p* = 0.021	1.233 ± 0.008 *p* = 0.051	5.76 ± 0.38 *p* = 0.395
11-MIX ^4^	511 ± 11 *p* = 0.001	1.037 ± 0.014 *p* < 0.001	1335 ± 22 *p* < 0.001	1.317 ± 0.012 *p* = 0.092	1847 ± 29 *p* < 0.001	1.215 ± 0.008 *p* < 0.001	6.22 ± 0.38 *p* = 0.720
12-OA ^4^	494 ± 11 *p* = 0.078	1.038 ± 0.014 *p* < 0.001	1287 ± 22 *p* = 0.034	1.324 ± 0.012 *p* = 0.252	1782 ± 29 *p* = 0.017	1.223 ± 0.008 *p* = 0.001	5.62 ± 0.38 *p* = 0.222
13-OA ^4^	501 ± 11 *p* = 0.019	1.028 ± 0.014 *p* < 0.001	1318 ± 22 *p* < 0.001	1.311 ± 0.012 *p* = 0.031	1820 ± 29 *p* < 0.001	1.208 ± 0.008 *p* < 0.001	6.05 ± 0.38 *p* = 0.918
14-OA ^4^	465 ± 11 *p* = 0.423	1.108 ± 0.014 *p* = 0.469	1266 ± 22 *p* = 0.237	1.340 ± 0.012 *p* = 0.884	1730 ± 29 *p* = 0.567	1.263 ± 0.008 *p* = 0.058	5.54 ± 0.38 *p* = 0.147
15-MIX ^4^	476 ± 11 *p* = 0.845	1.097 ± 0.014 *p* = 0.939	1278 ± 22 *p* = 0.085	1.338 ± 0.012 *p* = 0.977	1754 ± 29 *p* = 0.165	1.255 ± 0.008 *p* = 0.344	5.18 ± 0.38 *p* = 0.017
16-PRE ^4^	485 ± 11 *p* = 0.352	1.085 ± 0.014 *p* = 0.346	1304 ± 22 *p* = 0.004	1.335 ± 0.012 *p* = 0.822	1788 ± 29 *p* = 0.009	1.251 ± 0.008 *p* = 0.669	4.76 ± 0.38 *p* < 0.001
17-MIX ^4^	458 ± 11 *p* = 0.157	1.105 ± 0.014 *p* = 0.588	1228 ± 22 *p* = 0.593	1.354 ± 0.012 *p* = 0.185	1685 ± 29 *p* = 0.316	1.270 ± 0.008 *p* = 0.004	6.33 ± 0.38 *p* = 0.518
18-PFA ^4^	491 ± 11 *p* = 0.132	1.067 ± 0.014 *p* = 0.025	1226 ± 22 *p* = 0.524	1.353 ± 0.012 *p* = 0.216	1717 ± 29 *p* = 0.926	1.243 ± 0.008 *p* = 0.552	5.31 ± 0.38 *p* = 0.040
19-PRE ^4^	485 ± 11 *p* = 0.371	1.078 ± 0.014 *p* = 0.158	1229 ± 22 *p* = 0.624	1.336 ± 0.012 *p* = 0.850	1713 ± 29 *p* = 0.971	1.237 ± 0.008 *p* = 0.156	6.12 ± 0.38 *p* = 0.918
20-PFA ^4^	486 ± 11 *p* = 0.301	1.091 ± 0.014 *p* = 0.590	1228 ± 22 *p* = 0.592	1.334 ± 0.012 *p* = 0.748	1713 ± 29 *p* = 0.987	1.242 ± 0.008 *p* = 0.428	4.95 ± 0.38 *p* = 0.003
21-PFA ^4^	485 ± 11 *p* = 0.330	1.077 ± 0.014 *p* = 0.128	1246 ± 22 *p* = 0.799	1.321 ± 0.012 *p* = 0.170	1730 ± 29 *p* = 0.566	1.226 ± 0.008 *p* = 0.004	4.59 ± 0.38 *p* < 0.001
22-MIX ^4^	486 ± 11 *p* = 0.301	1.089 ± 0.014 *p* = 0.502	1270 ± 22 *p* = 0.179	1.320 ± 0.012 *p* = 0.146	1755 ± 29 *p* = 0.147	1.231 ± 0.008 *p* = 0.028	5.56 ± 0.38 *p* = 0.168
23-MIX ^4^	484 ± 11 *p* = 0.355	1.083 ± 0.014 *p* = 0.273	1251 ± 22 *p* = 0.612	1.327 ± 0.012 *p* = 0.378	1736 ± 29 *p* = 0.448	1.235 ± 0.008 *p* = 0.090	5.94 ± 0.38 *p* = 0.694
24-MIX ^4^	494 ± 11 *p* = 0.078	1.086 ± 0.014 *p* = 0.386	1292 ± 22 *p* = 0.018	1.307 ± 0.012 *p* = 0.014	1786 ± 29 *p* = 0.011	1.222 ± 0.008 *p* < 0.001	5.30 ± 0.38 *p* = 0.037
25-MIX ^4^	464 ± 11 *p* = 0.394	1.105 ± 0.014 *p* = 0.610	1255 ± 22 *p* = 0.489	1.311 ± 0.012 *p* = 0.028	1720 ± 29 *p* = 0.844	1.229 ± 0.008 *p* = 0.013	6.33 ± 0.38 *p* = 0.518
ICC ^5^	0.35	0.61	0.43	0.35	0.42	0.28	0.08

^1^ Results are reported as means ± standard deviation. Body weight gain (BWG) in grams/chicken, feed conversion ratio (FCR) in grams feed intake/grams weight gain and *Clostridium perfringens* (CP) counts as log_10_ colony forming units/gram cecal content. ^2^ Negative control (no feed additive); production performance data based on *n* = 66 pens, and CP data based on *n =* 198 individual chicken samples. ^3^ Narasin; production performance data based on *n* = 66 pens, and CP data based on *n =* 198 individual chicken samples. ^4^ Probiotics (PRO), prebiotics (PRE), phytogenics (PFA), organic acids (OA), mixed products (MIX); production performance data based on *n* = 11 pens, and CP data based on *n =* 33 individual chicken samples. ^5^ Intraclass correlation coefficient.
